# Preclinical evaluation of cyclin dependent kinase 11 and casein kinase 2 survival kinases as RNA interference targets for triple negative breast cancer therapy

**DOI:** 10.1186/s13058-015-0524-0

**Published:** 2015-02-11

**Authors:** Betsy T Kren, Gretchen M Unger, Md J Abedin, Rachel I Vogel, Christine M Henzler, Khalil Ahmed, Janeen H Trembley

**Affiliations:** 1 Research Service (151), Minneapolis VA Health Care System, One Veterans Drive, Minneapolis, MN 55417 USA; 2 Department of Laboratory Medicine and Pathology, University of Minnesota, 420 Delaware Street, SE, Minneapolis, MN USA; 3 Masonic Cancer Center, University of Minnesota, 717 Delaware Street SE Room 130, Minneapolis, MN 55414 USA; 4 GeneSegues Inc, 3180 High Point, Chaska, MN 55318 USA; 5 Minnesota Supercomputing Institute, University of Minnesota, 117 Pleasant Street SE, Minneapolis, MN 55455 USA; 6 Department of Urology, University of Minnesota, 420 Delaware St. SE, Minneapolis, MN 55455 USA

## Abstract

**Introduction:**

Targeted therapies for aggressive breast cancers like triple negative breast cancer (TNBC) are needed. The use of small interfering RNAs (siRNAs) to disable expression of survival genes provides a tool for killing these cancer cells. Cyclin dependent kinase 11 (CDK11) is a survival protein kinase that regulates RNA transcription, splicing and mitosis. Casein kinase 2 (CK2) is a survival protein kinase that suppresses cancer cell death. Eliminating the expression of these genes has potential therapeutic utility for breast cancer.

**Methods:**

Expression levels of CDK11 and CK2 mRNAs and associated proteins were examined in breast cancer cell lines and tissue arrays. RNA expression levels of *CDC2L1*, *CDC2L2*, *CCNL1*, *CCNL2*, *CSNK2A1*, *CSNK2A2*, and *CSNK2B* genes in breast cancer subtypes were analyzed. Effects following transfection of siRNAs against CDK11 and CK2 in cultured cells were examined by viability and clonal survival assays and by RNA and protein measures. Uptake of tenfibgen (TBG) nanocapsules by TNBC cells was analyzed by fluorescence-activated cell sorting. TBG nanocapsules delivered siRNAs targeting CDK11 or CK2 in mice carrying TNBC xenograft tumors. Transcript cleavage and response parameters were evaluated.

**Results:**

We found strong CDK11 and CK2 mRNA and protein expression in most human breast cancer cells. Immunohistochemical analysis of TNBC patient tissues showed 100% of tumors stained positive for CDK11 with high nuclear intensity compared to normal tissue. The Cancer Genome Atlas analysis comparing basal to other breast cancer subtypes and to normal breast revealed statistically significant differences. Down-regulation of CDK11 and/or CK2 in breast cancer cells caused significant loss of cell viability and clonal survival, reduced relevant mRNA and protein expression, and induced cell death changes. TBG nanocapsules were taken up by TNBC cells both in culture and in xenograft tumors. Treatment with TBG- siRNA to CDK11 or TBG- siRNA to CK2αα’ nanocapsules induced appropriate cleavage of CDK11 and CK2α transcripts in TNBC tumors, and caused MDA-MB-231 tumor reduction, loss of proliferation, and decreased expression of targeted genes.

**Conclusions:**

CDK11 and CK2 expression are individually essential for breast cancer cell survival, including TNBC. These genes serve as promising new targets for therapeutic development in breast cancer.

## Introduction

Targeted therapies for hormone receptor expression positive and for human epidermal growth factor receptor 2 (HER2, also known as ERBB2 or EGFR2) overexpression-positive disease have improved breast cancer mortality; however, breast cancer lacking these receptors, termed triple negative breast cancer (TNBC), presents particular challenges because of its highly aggressive nature. Given the need for new approaches to treat TNBC, we investigated the effectiveness of downregulation of the essential protein kinases cyclin-dependent kinase (CDK) 11 and casein kinase 2 (CK2) using RNA interference (RNAi) for killing this aggressive form of breast cancer. When targeting a survival gene, an RNAi or small interfering RNA (siRNA) approach to downregulate or eliminate the survival protein expression, and thus its function, has advantages of great flexibility and specificity in choosing the target. The difficulty in such an approach when moving to systemic organismal use comes with delivery of the nucleic acids in a protected and tumor-directed manner. We have developed tenfibgen (TBG) nanoencapsulation technology that allows for delivery of nucleic acids into malignant cells *in vivo* while avoiding accumulation in normal cells [1-3].

The first CDK family members characterized were the catalytic subunits that formed heterodimers with regulatory partner proteins, called cyclins. The prototypical CDKs (such as CDK1 and CDK2) displayed cell cycle phase-specific activity; however, there are now members of the CDK family that play more varied roles in cellular regulation [4,5]. CDK11 (formerly named PITSLRE) is a somewhat atypical CDK that is essential for cell survival [6,7]. CDK11 is evolutionarily well conserved with two almost identical CDK11 genes in humans (*CDC2L1* and *CDC2L2*) and one CDK11 gene in other organisms, including mice [8]. Cyclin L1 (*CCNL1*) and cyclin L2 (*CCNL2*) are confirmed regulatory partner proteins for CDK11 [9-14]. Expression of both CDK11 and cyclin L1 is increased in various cancers, with amplification of *CCNL1* associated with poor prognosis [15-20]. Transcription and alternative splicing generate more than 20 distinct CDK11 mRNA and protein isoforms in human cells, and the alternative splicing involves exons encoding the N-terminal domain, but not exons in the C-terminal kinase catalytic domain [8]. Gene mutation does not play a significant role in CDK11 function in cancer, and the majority of mutations reported are missense, suggesting again the essential nature of CDK11 function (Sanger COSMIC database).

The predominant CDK11 protein isoforms during cell proliferation are designated p110 and p58 for their respective observed mass by polyacrylamide gel electrophoresis (CDK11^p110^, CDK11^p58^). The CDK11^p110^ protein isoforms are ubiquitously expressed in mammalian tissues and cell lines during proliferation and throughout the cell cycle [21]; moreover, CDK11^p110^ continues to be detected by immunoblot in quiescent mouse liver [9]. The p110 isoforms associate with multiple transcription and splicing related proteins via the N-terminal (nonkinase) domain and have been shown to influence transcription and splicing activities [9,22-28]. The CDK11^p58^ isoforms are translated at the G2/M cell cycle transition from an internal ribosomal entry site on the same mRNA transcripts that produce the p110 isoforms [29]. CDK11^p58^ is only produced during a very narrow window, and is therefore difficult to detect in unsynchronized cells. CDK11^p58^ is necessary for successful mitosis and is involved with centrosome maturation, bipolar spindle formation, and centriole duplication [6,30-35].

The CK2 (formerly casein kinase II) enzyme is a well-established cancer target with a heterotetrameric composition of two catalytic α and/or α' subunits (42 and 38 kDa, respectively) joined together by two β subunits (28 kDa). CK2 phosphorylates a vast number of substrates, thereby influencing a multitude of cellular processes [36]. CK2 does not require specific activation, and thus generally exhibits constitutive activity in cells with minor fluctuations in expression or activity from various signaling inputs [37-40]. CK2 is ubiquitous, its steady-state levels are distinct for various cell types, and its expression in different tissues under normal conditions remains constant. However, increased CK2 protein expression and activity is observed in a broad range of cancers including breast cancer, correlates with increased nuclear localization, is not associated with any mutational changes in CK2 genes, and serves as a prognostic indicator [41-49]. Notably, expression of CK2α in the mouse mammary gland under control of the mouse mammary tumor virus long terminal repeat (MMTV-LTR) gave rise to a transgenic mouse model of breast cancer [50]. Finally, suppression of apoptosis by CK2 supports its role in cell survival, thus decisively connecting CK2 upregulation and function to the cancer cell phenotype [42,51].

Owing to the essential roles for CDK11 and CK2 in both cycling and noncycling cells, tumor-targeted delivery of a therapeutic is important. To accomplish protected tumor cell-specific *in vivo* delivery of siRNAs, the nucleic acid is condensed and coated with a TBG protein shell that is recognized by tenascin-C receptors [2,52,53]. Tenascin-C-rich stroma has been observed consistently adjacent to the epithelial cell nests of breast [54] and other cancers; further, tenascin-C appears to play a role in initiating and sustaining lung colonization of breast cancer cells [55]. TBG nanocapsule cellular uptake utilizes a lipid raft-mediated caveolar pathway [1,56]. The TBG-based nanocapsules containing siRNA average 10 to 30 nm in size, display a slight negative charge, and exist as uniform nonaggregated particles in solution. The TBG-siCK2 nanocapsule has been shown to be specific for malignant and not normal prostate cells in culture, and a similar TBG-RNAi-CK2 nanocapsule has been shown to be specific for malignant and not normal tissues in mice [1-3].

Our data presented herein demonstrate that both CDK11 and CK2 kinases are well expressed in breast cancer cells and are essential for cell survival. TBG nanocapsules enter human TNBC cells, both in cell culture and when systemically administered to mice carrying xenograft tumors. TBG-siCDK11-treated or TBG-siCK2-treated MDA-MB-231 tumors showed reduced target protein expression and response parameters that included decreased proliferation, tumor mass, and tumor volume relative to controls.

## Materials and methods

### Cell lines and culture

MCF-10A, MCF-12A, MDA-MB-468, and BT-474 cells were obtained from ATCC (Manassas, VA, USA). MDA-MB-231 and MCF-7 cells were a gift from Dr David Potter (University of Minnesota, MN, Minneapolis, USA). Tamoxifen-resistant MCF-7 L (MCF-7L^TamR^) cells were a gift from Dr Douglas Yee (University of Minnesota). SUM-149 and SUM-190 cells were a gift from Dr Sofia Merajver (University of Michigan, MI, Ann Arbor, USA). Growth conditions include: MDA-MB-231 in Dulbecco’s modified Eagle’s medium (DMEM)/F12 (SH30023; HyClone Logan, Utah, USA), 10% fetal bovine serum (FBS), 1% penicillin–streptomycin; MCF-7 in phenol-free DMEM/F12 (SH30272; Hyclone), 10% charcoal-stripped FBS, 10 μg/ml bovine insulin, 1% penicillin–streptomycin; MCF-10A in DMEM/F12, 5% horse serum, 20 ng/ml human epidermal growth factor, 100 ng/ml cholera toxin, 10 μg/ml bovine insulin, 500 ng/ml hydrocortisone; MCF-12A in DMEM/F12, 5% horse serum, 20 ng/ml human epidermal growth factor, 100 ng/ml cholera toxin, 10 μg/ml bovine insulin, 500 ng/ml hydrocortisone; MDA-MB-468 in DMEM (11995; Gibco Carlsbad, CA, USA), 10% FBS, 1% penicillin–streptomycin; MCF-7 L/Tam^R^ in phenol-free DMEM/F12, 5% charcoal-stripped FBS, 5 μg/ml bovine insulin, 1% penicillin–streptomycin; SUM-149 in Ham’s F12 (11765; Gibco), 5% FBS, 5 μg/ml bovine insulin, 1 μg/ml hydrocortisone, 0.5 μg/ml amphotericin B, 5 μg/ml gentamycin; and SUM-190 in Ham’s F12, 0.5 g/l bovine serum albumin, 5 mM ethanolamine, 10 mM HEPES, 5 μg/ml transferrin, 10 nM 3,3′,5-triiodo-l-thyronine, 50 nM sodium selenite, 5 μg/ml bovine insulin, 1 μg/ml hydrocortisone, 0.5 μg/ml amphotericin B, 5 μg/ml gentamycin. Cells were grown in an incubator at 37°C with 5% carbon dioxide_,_ with the exception of the SUM cell lines that were grown with 10% carbon dioxide. All cells had undetectable levels of mycoplasma when thawed, and were maintained in culture for up to 3 months. The MDA-MB-231, SUM-149, SUM-190, MCF-7, and MCF-7L^TamR^ cell lines were authenticated by STR profiling (IDEXX BioResearch, Columbia, MO, USA).

### Oligonucleotides

Standard chemistry siRNAs were obtained from Dharmacon (Lafayette, CO, USA). The sense strand sequences are: siCK2, 5′-auacaacccaaacuccacauuudTdT-3′ [2]; siCDK11, 5′-gagcgagcagcagcgugugdTdT-3′ [30]; and siCON1, 5′-uagcgacuaaacacaucaauudTdT-3′ [57].

### Immunofluorescence and immunohistochemical analyses

Ki-67 staining was performed by the Pathology and Laboratory Medicine Service (Minneapolis VA Health Care System, Minneapolis, MN, USA). Analysis of the Ki-67 staining was performed using the ImmunoRatio web application [58].

CDK11 staining was tested by immunofluorescence analysis in cultured cells and by immunohistochemical analysis in xenograft tumor tissues using anti-CDK11 rabbit polyclonal antibodies from Santa Cruz Biotechnology (PITSLRE sc-928; Santa Cruz, CA, USA) and from Bethyl Laboratories (A300-311A; Montgomery, TX, USA). A negative control section (without primary antibody) was included in the testing to verify the specificity of the signal. The Bethyl Laboratories antibody was chosen for immunofluorescence work and the Santa Cruz antibody was chosen for immunohistochemical work.

For immunofluorescence analyses, breast cancer cells were plated onto glass coverslips precoated with either poly-d-lysine at 0.5 mg/ml (P6407; Sigma St. Louis, MO, USA) or with Matrigel at 0.33 mg/ml (354234; BD Biosciences San Jose, CA, USA). Next day, the cells were fixed in 2% paraformaldehyde for 15 minutes at room temperature, washed three times in phosphate-buffered saline (PBS), incubated for 30 minutes in glycine (100 mM, pH 7.4), and washed three times in PBS. The fixed cells were permeabilized with 0.1% Triton X-100/PBS for 3 minutes, and then washed three times in PBS. Cells were incubated with Sea Block containing 6 mg/ml normal donkey for 30 minutes at room temperature, washed once in PBS, then incubated with CDK11 antibody (A300-311A, 2 μg/ml; Bethyl Laboratories) in a humidified container at 37°C for 1 hour. Cells were washed three times for 5 minutes in PBS containing 0.05% Igepal CA-630 (Sigma) and incubated with secondary antibody (donkey anti-rabbit F(ab′)2 DyLight-488 711-486-152, 0.75 mg/ml in 50% glycerol, 1:400; Jackson Immunoresearch, West Grove, PA, USA) in a humidified container at 37°C for 1 hour. Cells were washed six times for 5 minutes in PBS containing 0.05% Igepal CA-630 and counterstained using Slowfade Gold containing DAPI (S36938; Invitrogen Carlsbad, CA, USA). Images were captured using an Olympus BX60 (Shinjuku, Toyko, Japan) fluorescent microscope at 40× objective with a digital color Q Imaging Retiga 2000R Fast1394 camera (Surrey, British Columbia, Canada).

CDK11 immunohistochemical staining was performed on the following commercially purchased arrays (US Biomax, Inc. Rockville, MD, USA): T085, T088a; BR243f, BR487 (TNBC); and BR1503b. The following statement was taken from the US Biomax website: ‘All tissue is collected under the highest ethical standards with the donor being informed completely and with their consent. We make sure we follow standard medical care and protect the donors’ privacy. All human tissues are collected under HIPPA approved protocols’. This work was performed under Minneapolis VA Health Care System Subcommittee on Research Safety protocol number 130601. Slides were deparaffinized by three changes of xylene (10 minutes each). The sections were then rehydrated by passing through graded ethanol (100% × 10 minutes, 100% × 5 minutes, 95% × 5 minutes, 80% × 5 minutes). Heat-induced antigen retrieval was performed with a citrate-based antigen retrieval solution, pH 6.0 (Rodent Decloaker, Biocare Medical, CA, Concord, USA) for 30 minutes. The endogenous tissue peroxidase activity was quenched with a 3 to 5% hydrogen peroxide solution in PBS for 10 minutes. The tissue slides were blocked with Background Sniper Reagent (Biocare Medical) in 5% skim milk for 30 to 60 minutes. The slides were incubated with primary CDK11 antibody (1:50, sc-938; Santa Cruz, CA, USA) overnight at 4°C. The antigen–antibody complex was detected using Rabbit-on-Rodent-HRP-Polymer for 30 minutes and the betazoid 3,3′-diaminobenzidine peroxidase chromogenic kit (Biocare Medical). Hematoxylin (Vector Lab Burlingame, CA, USA) was used as nuclear counterstain and washed in Clarifier 2 and Bluing reagent (Thermo Scientific Waltham, MA, USA) to obtain better cytoplasm to nuclear contrast. The slides then underwent dehydration processing using graded ethanol, and were washed with xylene before addition of a coverslip with nonaqueous permanent mounting medium (Permount; Fisher Scientific Waltham, MA, USA). Images were captured in bright field as described above.

Scoring for CDK11-positive staining was performed by two independent observers and for each tissue represented the mean of the two observers’ scores. CDK11 staining was given an intensity rating (0, no stain; 1+, faint nuclear stain; 2+, moderate nuclear stain; 3+, strong nuclear stain) and a distribution rating representing the percentage of positive cells (0, no positives; 1, <10% positive; 2, 10 to 50% positive; 3, >50% positive). The final score per tissue section was the product of the intensity and distribution ratings.

### Cell viability assays

Transfection complexes were formed combining 30 nM single siRNA or 15 nM each of combined siRNAs with 10 μl Dharmafect 1 or 2 and OPTI-MEM in a total volume of 400 μl. After a 20-minute incubation, 1.6 ml antibiotic-free media containing 10% FBS was mixed in and the total volume added to cells at 50% confluence on 60 mm plates. After 5 hours of incubation at 37°C/5% carbon dioxide, an additional 2 ml complete media were added to each plate. Next day, the cells were trypsinized and cells were plated (8,000 cells for MDA-MB-231 and SUM-149; 10,000 cells for MCF-7L^TamR^) into four wells per transfection condition in Primaria 96-well plates coated with 0.33 mg/ml Matrigel (354234; BD Biosciences), 0.2 μg/ml tenascin-C (CC065; Millipore Billerica, MA, USA) and 0.1 μg/ml fibronectin (F-0895; Sigma). Ninety-six hours following transfection initiation, CellTiter 96® Aqueous One Assays were performed according to the manufacturer’s instructions (Madison, WI, USA). Absorbance values for media alone were subtracted from the experimental values. The data shown represent the results of five experiments for MDA-MB-231, of four experiments for SUM-149, and of two experiments for MCF-7^TamR^.

### Clonal survival assays

Cells were transfected as described above, and 48 hours after the first transfection the cells were replated for 50 to 60% confluence the next day and transfected a second time. Twenty-four hours after the second transfection, the cells were collect using trypsin and plated in triplicate at a concentration of 2,000 cells per 35 mm plate in standard media. The media were replaced after 4 days, and 7 days after plating the cells were stained with crystal violet for 20 minutes (1× PBS containing 1% (v/v) methanol, 1% (v/v) formaldehyde and 0.05% (w/v) crystal violet), the stain removed, and plates washed by immersion in water with continuous water flow. Plates were air-dried, colonies containing at least 50 cells were counted, and plates were scanned. The data shown represent the results of three replicates per experiment with three experiments performed per cell line.

### Immunoblot analysis

Cell pellets from cultured cells were processed in radioimmunoprecipitation assay buffer and 20 μg subjected to 8%, 10% or 12% Tris–glycine SDS-PAGE as described previously [59]. For lysates from tumors, approximately 0.1 g frozen tumor tissue was minced and homogenized on ice in 1 ml CSK buffer (10 mM PIPES pH 6.8, 300 mM sucrose, 3 mM MgCl_2_, 1 mM ethylenediamine tetraacetic acid (EDTA), 0.5% Triton X-100) and centrifuged for 15 minutes at 600 × *g* at 4°C. This processing resulted in extraction of the nuclear proteins into the supernatant (verified by immunoblot results). The resulting supernatant was quantitated using the Bradford assay (23238; Thermo Scientific), and 30 μg each lysate were separated using the NuPAGE 4 to 12% Bis–Tris and Novex 4 to 12% Tris–Glycine Midi gel systems (Life Technologies Waltham, MA, USA). The membranes were blocked for 30 minutes with 5% nonfat milk (170–6404; Bio-Rad Hercules, CA, USA) or 5% bovine serum albumin (A-9647; Sigma) in Tris-buffered saline (pH 7.4) with 0.1% Tween 20 at room temperature. Antibodies were diluted into fresh blocking buffer according to the manufacturer’s recommendations, and the membranes processed as described [59]. Antibodies used were: CDK11 (A300-311A), cyclin L1 (A302-058A), CK2α (A300-197A) and CK2α′ (A300-199A) from Bethyl Laboratories; cyclin L2 (600-401-878) from Rockland Immunochemicals (Limerick, PA, USA); CK2β (sc-12739 and sc-46666) and actin (sc-1616) from Santa Cruz Biotechnology; CDK11 (5524), caspase 3 (9661, 9662), lamin A/C (2032), and Bcl-xL (2762) from Cell Signaling (Beverly, MA, USA); and survivin (AF886) from R&D Systems (Minneapolis, MN, USA).

### Quantitative real-time RT-PCR analysis

Total RNA was isolated from frozen cell pellets using the RNeasy mini kit (Qiagen Valencia, CA, USA), including the on-column DNase digestion according to the manufacturer’s protocol, and quantitated using a NanoDrop spectrophotometer. The Superscript III kit (Invitrogen) was used to synthesize cDNA from total RNA (0.25 μg) using oligo-dT primers according to the manufacturer’s protocol. FAM TaqMan gene expression probes hs00751002_s1 (CK2α), hs00176505_m1 (CK2α′), hs00414449_m1 (CDK11), and hs01003267_m1 (HPRT-1), TaqMan Fast 2X Mastermix and UNG amperase were from Applied Biosystems, Inc. (Foster City, CA, USA). Reactions were run according to manufacturer’s specification using 96-well FAST plates on an ABI 7900HT machine (Applied Biosystems, Inc.). Analyses were performed using the SDS 2.3 ABI software (Applied Biosystems, Inc.) and changes calculated according to the 2^(−∆∆Ct)^ method. HPRT-1 was used as the reference gene for normalization. All results are reported as the average of reactions run in duplicate.

### Nanocapsule preparation

For TBG nanocapsules, a dispersion atomization method was used to package siRNA oligonucleotides into nanocapsules composed of TBG. The modified method of Aukhil and colleagues was used to prepare TBG [60]. All other reagents used were of the highest purity available. Briefly, 250 μg siRNA was complexed with 37.5 μg spermine (0.5 μg/μl; Sigma-Aldrich St. Louis, MO, USA) and dispersed using a water-insoluble surfactant system consisting of 10 μg 2,4,7,9-tetramethyl-5-decyn-4,7-diol (50% v/v) in dimethylsulfoxide (Air Products and Chemicals, Inc. Allentown, PA, USA). Following emulsification into a water-insoluble surfactant dispersed into a water-miscible solvent (dimethylsulfoxide), the siRNA was then inverted by dilution into suspension with addition of 750 μl sterile PBS, pH 7.2. The resultant hydrophobic micelles were coated by adsorption to 12.5 μg TBG (1 μg/μl) dispersed into the solution prior to spray dispersion atomization into a 25 ml LiCl salt solution (135 mM Li^+^, 9 mM Ca^2+^, 37.5 nM Sr^2+^, 12.5 nM Mg^2+^). Following incubation at 4 to 6°C with rotation in the salt solution for more than 14 hours, the nanocapsules were recovered by centrifugation at 20,000 x *g* for 2 hours, and resuspended in PBS containing 10% lactitol (w/v) at a theoretical concentration of 1 μg/μl for 0.2 μm filter sterilization prior to characterization. Average particle diameters were measured from transmission electron microscopy images. Surface charge determinations were confirmed by published methods [61].

### Fluorescence-activated cell sorting analysis of cultured cells and xenograft tumors

MDA-MB-231 (4 × 10^5^) cells and SUM-149 (8 × 10^5^) cells were plated onto 60 mm plates precoated overnight with 0.25 μg/ml of 3:1 tenascin-C (CC065; Millipore)/fibronectin-1 (F089; Sigma). The media for MDA-MB-231 cells included 12 μl/ml human low-density lipoprotein to improve nanocapsule uptake. The next morning, media were replaced with 2 ml media containing 125 ng/ml TBG-dysprosium (Dy). For MDA-MB-231 cells that were treated twice with TBG-Dy, the media were again replaced with 2 ml media containing 125 ng/ml TBG-Dy 12 hours after the first treatment. The following morning, all cells were 70 to 90% confluent and were collected and subjected to fluorescence-activated cell sorting (FACS) analysis. A minimum of 10,000 events from each cell transfection or untreated cells were collected. TBG-Dy uptake by cells was determined by gating for Dy fluorescent signal (em 625, Per-CP/Cy5.5 filter). Data were collected and analyzed using a FACS ARIA III (Becton Dickinson San Jose, CA, USA) and FACS DIVA software version 6.0 (BD Biosciences San Jose, CA, USA).

Mice carrying MDA-MB-231 or SUM-149 xenograft tumors initiated in the mammary pad (~300 mm^3^) were injected with 200 nmol/kg TBG-Dy by tail vein. Next day, the tumors were collected on ice, finely minced, and incubated at 37°C in 1× collagenase/hyaluronidase (36254; StemCell Tech Vancouver, British Columbia, Canada) in DMEM/F12 with 5% FBS at a ratio of 10 ml solution per 1 g tumor. Digestion mixtures were mixed using a transfer pipette every 15 minutes. After 60 minutes, an equal volume of Hank’s balanced salt solution (SH30031.02; HyClone) with 2% FBS was added and the cells centrifuged 350 × *g* for 5 minutes. Cells were resuspended in 2 ml of 0.05% trypsin/0.025 mM EDTA and incubated for 5 minutes at room temperature. The digestion was stopped by addition of 3 ml DMEM/F12 with 5% FBS and centrifugation at 350 × *g* for 5 minutes. The cells were resuspended and continuously pipetted for 1 minute in 2 ml dispase (07913; StemCell Tech) prewarmed to 37°C containing 0.1 ml of 1 mg/ml DNase I (07900; StemCell Tech). Ten milliliters of ice-cold Hank’s balanced salt solution with 2% FBS were added to the digest, and the cells centrifuged at 350 × *g* for 5 minutes. Cells were resuspended in 5 ml red blood cell lysis buffer (150 mM ammonium chloride, 10 mM potassium bicarbonate, 0.1 mM EDTA), followed by 20 ml ice-cold PBS, and centrifuged at 350 × *g* for 5 minutes. Cells were resuspended in 5 ml ice-cold Hank’s balanced salt solution with 2% FBS, passed sequentially through 100 μm and 40 μm cell strainers and centrifuged at 350 × *g* for 5 minutes. The final cell pellet was resuspended in 2 ml DMEM. FACS analysis was performed as above on the tumor cell suspensions collecting 100,000 events.

### 5′ RNA ligase-mediated RACE

MDA-MB-231 xenograft and SUM-149 mammary pad tumors from different acute dosing experiments were used for total RNA isolation using TRIzol (Life Technologies), and the quality of RNA was verified by gel electrophoresis. Total RNA (10 μg) from the tumors was ligated with 0.5 μg RNA adaptor oligomer (5′-cgacuggagcacgaggacacugacauggacugaaggaguagaaa-3′) that contains the forward 5′ adaptor primer binding site in a 20 μl reaction using 20 U RNA ligase (New England Biolabs Ipswich, MA, USA) and 40 U RNaseOUT™ (Life Technologies) according to the manufacturer’s recommended conditions. The ligated RNA was purified by diafiltration (Ultracel-30 K; Millipore) using conditions for nucleic acids outlined by the manufacturer, and the quality of the ligated RNA product was verified by gel electrophoresis. Eight microliters of the ligated RNA product were reverse transcribed using Superscript III (Life Technologies) and a CDK11 gene-specific primer (5′-ACAAAGTAAGACGAGGAGTTCCGAG-3′), a CK2α gene-specific primer (5′-CGCTTTCGAGAGTGTCTGCCCAAGA-3′), or a CK2α′ gene-specific primer (5′-GGTGTCTGTTCTCACTATGG-3′) designed to hybridize 3′ to the predicted RNAi-mediated cleavage sites in the respective transcripts. The resulting cDNAs (2 μl) were used for PCR using the forward RNA adaptor primer (5′-GGACACTGACATGGACTGAAGGAGTA-3′) and the reverse CDK11 (5′-TGGTGGTAAGGTGGAAGCCCGTCTC-3′), CK2α (5′-TCACTGTGGACAAAGCGTTCCCATC-3′), or CK2α′ (5′-TGGATAAAGTTTTCCCAGCG-3′) gene-specific primer. PCR was performed using the Expand High Fidelity system (Roche Applied Science Indianapolis, IN, USA) using dNTP, buffer and enzyme concentrations recommended by the manufacturer. PCR for CK2αα′ RACE products was performed using 95°C for 3 minutes, 40 cycles of amplification (94°C for 45 seconds, 57°C for 30 seconds, 72°C for 45 seconds), and 72°C for 10 minutes. CDK11 RACE products were amplified using 94°C for 2 minutes; five cycles of 94°C for 30 seconds and 72°C for 1 minute; 34 cycles of 94°C for 45 seconds, 65°C for 30 seconds, 72°C for 45 seconds; and 72°C for 10 minutes. PCR products were analyzed by 2% agarose Tris–borate–EDTA gel electrophoresis stained with ethidium bromide and visualized by UV light. Oligomers were obtained from Integrated DNA Technologies (Coralville, IA, USA).

### Mouse xenograft therapeutic acute effect studies

Female athymic NCr nude (Nu/Nu) mice (01B74; National Cancer Institute Frederick, MD, USA) were maintained under pathogen-free conditions. MDA-MB-231 tumors were initiated by subcutaneous injection of 2 × 10^6^ cells in 50% Matrigel (354234; BD Biosciences) in the mouse flank when mice were 7 to 8 weeks old. Therapy was initiated when tumors reached an average size of 150 mm^3^, calculated using the formula:

$$ \mathrm{Volume} = \left(\mathrm{length} \times \mathrm{width} \times \mathrm{width}\right)\ /\ 2 $$

Groups of mice (six to seven mice per group) were subjected to intravenous injection on days 1, 4 and 7 with 0.01 mg/kg TBG-siCDK11, TBG-siCK2, or TBG-siCON1 in Plasma-Lyte-A (Baxter Deerfield, IL, USA). Mice were sacrificed on day 10, and the tumor, liver, spleen, kidney, ovary, and normal mammary pad were excised, weighed, and snap frozen in liquid nitrogen for protein analysis or were placed in formalin. The animal facilities were approved by the Association for the Assessment and Accreditation of Laboratory Animal Care International and all animal research was performed in strict accordance with the recommendations in the Guide for the Care and Use of Laboratory Animals and with the current regulations and standards of the USDA, the US Department of Health and Human Services, and the National Institutes of Health. Animal experiments were conducted in the Minneapolis VA Health Care System animal facility in accordance with a protocol approved by the Minneapolis VA Health Care System Institutional Animal Care and Use Committee (protocol number 130601).

### Image processing

Immunoblot images from the siRNA-transfectedcultured cells were adjusted in size to align lanes from different comb sizes. Black lines on immunoblots and white lines on agarose gels indicate removal of intervening lanes.

Adobe Photoshop adjustments were made. No gamma changes were made to any image. For immunofluorescence and transmission electron microscopy images, contrast was adjusted to +100. For immunohistochemistry (IHC) images, brightness was adjusted to +100 and contrast to +50.

### Statistical analysis

IHC scores for the CDK11 signal on human breast cancer arrays were determined by two independent observers. The average value was taken for each sample. The comparison between normal tissue and TNBC was conducted using a Wilcoxon rank-sum test and the median and range for each are reported. Mean immunoblot protein levels, cell viability values, and the number of clonal survival colonies were summarized and compared by treatment group using analysis of variance (ANOVA). The mean ± standard deviation is presented unless otherwise indicated. Mouse tumor volumes on days 7, 9 and 10 (relative to day 1) and primary tumor weights were summarized and compared by treatment group using ANOVA. *P* values for pairwise comparisons for the above analyses were conservatively adjusted for multiple comparisons using a Bonferroni correction. Differences between mouse weights on day 10 were compared by treatment group using paired *t* tests. The percentage of Ki67-positive cells was compared by treatment using ANOVA, adjusting for repeated measures within mice. Least-squares means ± standard errors are presented. *P* values for these comparisons with the control group were adjusted for multiple comparisons using Dunnett’s method.

TCGA breast cancer RSEM-normalized read count RNASeq (v.2, level 3) data were downloaded from The Cancer Genome Atlas data portal (November 2014) and associated metadata (PAM50 RNAseq calls, sample type data) were downloaded from the UCSC Cancer Genome Browser (November 2014). Log_2_-transformed RSEM normalized read counts were used as the final gene expression measurement. ANOVA and Student *t* tests were used to test for differences in expression for seven genes between basal breast cancer subtype and normal or other subtype primary tumors. *P* values for pairwise comparisons were conservatively adjusted for multiple comparisons using a Bonferroni correction. *P* <0.05 values are reported and considered statistically significant for all analyses.

## Results

### Expression of CDK11 and CK2 protein complex constituents in breast cancer cells

**Figure 1:**
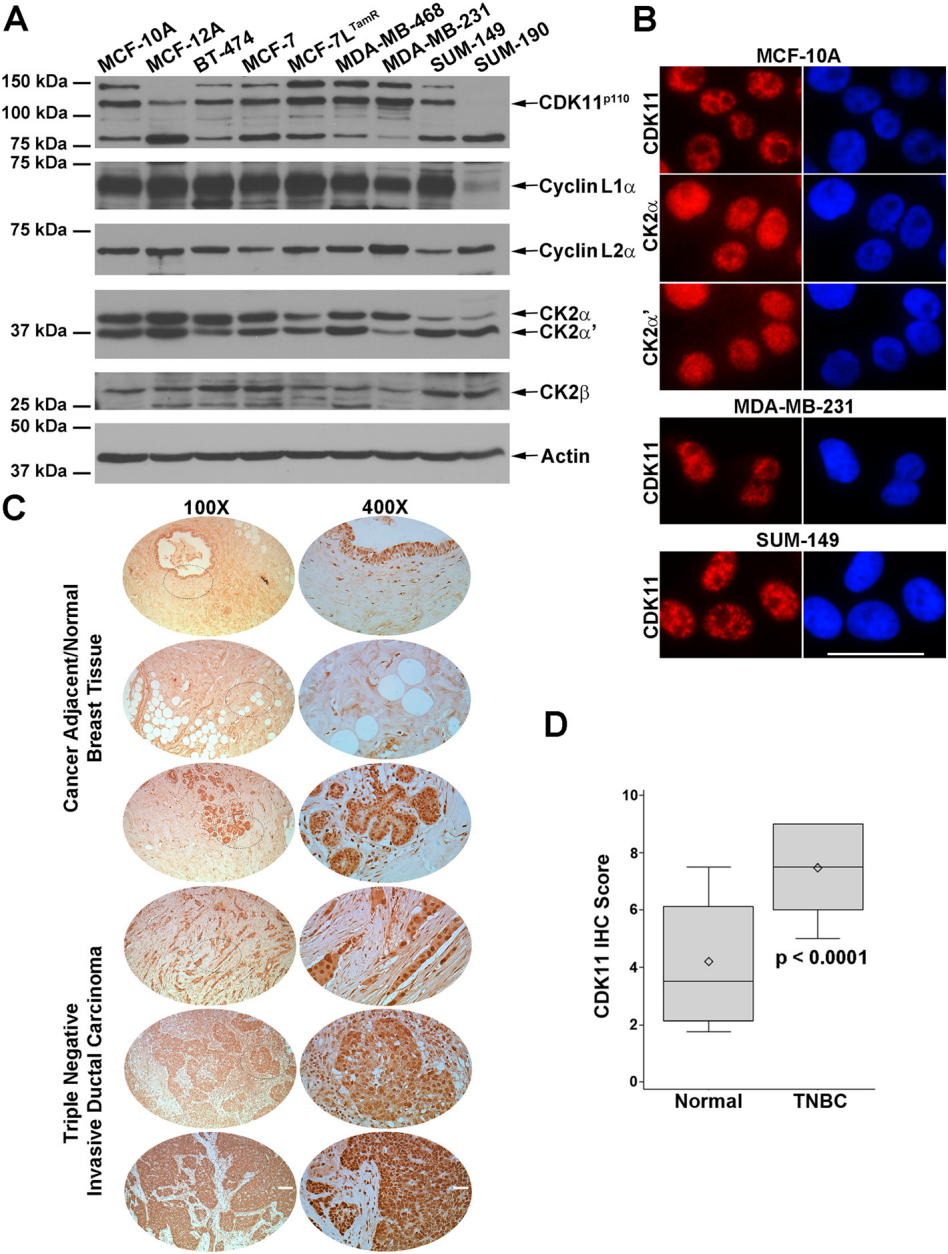
**Expression of CDK11 and CK2 protein complex members in untransformed and malignant breast cells. (A)** Immunoblot analysis of cultured breast cell lines, as indicated above the blots. Proteins detected are indicated on the right side of the blots. Actin signal was used as the loading control. **(B)** Indirect immunofluorescent detection of CDK11, CK2α, and CK2α′ (red color) in breast cell lines. Cell lines are indicated above each set of images and proteins detected are indicated on the left side of the images. Blue, 4′,6-diamidino-2-phenylindole-stained nuclei. Scale bar: 100 μm. **(C)** Immunohistochemical detection of CDK11 proteins in human normal and malignant breast tissue. Type of breast tissue indicated on the left side of the images. Magnification indicated above the images; dotted ellipse, portion of the 100× image that is shown at 400×. Scale bars: 400 μm for 100× and 100 μm for 400× images. **(D)** Human microarray tissues stained for CDK11 were scored by two independent observers. The average value was taken and the results plotted for normal (*n* = 16) versus triple-negative breast cancer (TNBC; *n* = 44) tissues. Box, first to third (Q1 to Q3) quartiles; diamond, mean; line inside box, median; whiskers, minimum and maximums of data range. CDK, cyclin-dependent kinase; CK2, casein kinase 2.

We examined the steady-state protein expression levels for CDK11 and CK2 protein complex members in breast cancer cell lines representing a range of subtypes. We also included nontransformed, immortalized cell lines (see Table 1). CDK11^p110^ and partner cyclins L1α and L2α were well expressed in these cell lines, with the exception of SUM-190 (Figure 1A). CDK11^p110^ was detected in SUM-190 cells upon longer exposure. In these breast cancer cell lines, detection of CDK11 displayed the prominent CDK11^p110^ isoform along with lesser expression of slightly smaller alternatively spliced isoforms, as has been observed previously [8,9]. Cyclin L1α is detected as a doublet or triplet when using radioimmunoprecipitation assay buffer, which can be observed in SUM-190 lysate due to the lower expression level. Cyclin L2α was detected as a single band. CK2α and CK2α′ proteins were simultaneously detected and demonstrated strong expression levels. The expression level of CK2α′ relative to CK2α was unusually high in the inflammatory breast cancer cells, suggesting a possible role for CK2α′ in inflammatory breast cancer. CK2β was moderately expressed in all cell lines.

**Table 1:** **Characteristics of breast cancer cell lines**

**Cell line** ^**a**^	**Transformed**	**Molecular subtype**	**Histological pathology** ^**b**^	**ER expressed**	**PR expressed**	**Her2 amplified**
MCF-10A	No	Basal	Fibrocystic	No	No	No
MCF-12A	No	Basal	Fibrocystic	No	No	No
BT-474	Yes	Luminal B	DC	Yes	Yes	Yes
MCF-7	Yes	Luminal A	AC	Yes	Yes	No
MCF-7L^TamR^	Yes	Luminal A	AC	Yes	Yes	No
MDA-MB-231	Yes	Basal	AC	No	No	No
MDA-MB-468	Yes	Basal	AC	No	No	No
SUM-149	Yes	Basal	IBC	No	No	No
SUM-190	Yes	Basal	IBC	No	No	Yes

AC, adenocarcinoma; DC, ductal carcinoma; ER, estrogen receptor; IBC, inflammatory breast cancer; PR, progesterone receptor; Her2, human epidermal growth factor receptor 2. ^a^MCF-10A and MCF-12A are spontaneously immortalized cells. ^b^Original tissue or tumor.

Intracellular protein expression patterns for CDK11 and CK2αα′ in nontransformed MCF-10A cells were examined by indirect immunofluorescence (Figure 1B). CDK11 proteins were detected mainly in the nuclei with prominent transcription and pre-mRNA splicing speckle distribution and more diffuse nucleoplasmic localization, as has been seen in nonbreast cell lines [9]. CK2α and CK2α′ proteins were localized in both nuclei and cytoplasm with a much stronger, finely speckled nuclear signal than cytoplasmic signal. CDK11 protein localization in TNBC cells was also evaluated and found to mirror that observed in the nontransformed cells (Figure 1B).

### CDK11 and CK2 mRNA expression in triple-negative breast cancer cells

We investigated how transcript expression related to protein expression in the nontransformed and TNBC cells for CDK11, CK2α and CK2α′. Data from quantitative real-time RT-PCR are summarized in Table 2. In general, protein expression and mRNA expression levels were similarly related for the TNBC cells. For example, in MDA-MB-468 cells roughly equal amounts of CK2α and CK2α′ protein levels are detected (Figure 1A), and likewise roughly equal amounts of the corresponding mRNA transcripts are expressed. In the nontransformed MCF-10A and MCF-12A cells, equal amounts of CK2α and CK2α′ proteins are expressed but the relative amounts of their corresponding mRNAs differ. For CDK11, more mRNA is expressed in MDA-MB-231 and MDA-MB-468 cells than in SUM-149 cells, which matches the relative amounts of CDK11^p110^ protein detected. In contrast, the nontransformed MCF-10A and MCF-12A breast cells expressed roughly 1.6-fold greater CDK11 mRNA relative to MDA-MB-231 and MDA-MB-468, but expressed equal or lesser amounts of protein.

**Table 2:** **mRNA expression levels in nontransformed and malignant breast cells** ^**a**^

**Cell line**	**CDK11** ^**b**^	**CK2α**	**CK2α** **′**
MCF-10A	1.53 ± 0.04	2.90 ± 0.35	5.19 ± 0.36
MCF-12A	1.38 ± 0.07	1.91 ± 0.17	1.05 ± 0.03
MDA-MB-231	0.96 ± 0.10	2.91 ± 0.48	0.40 ± 0.004
MDA-MB-468	0.87 ± 0.11	3.18 ± 0.06	2.91 ± 0.39
SUM-149	0.31 ± 0.001	0.66 ± 0.08	1.84 ± 0.01

Data presented as mean ± standard error. ^a^mRNA expression normalized to HPRT-1. ^b^Both human genes (*CDC2L1* and *CDC2L2*) recognized by PCR probes.

### CDK11 proteins are highly expressed in human triple-negative breast cancer tissue

We also examined CDK11 protein expression in human normal and malignant breast tissue using tissue microarrays. The antibody used for detection of CDK11 in the tissues recognizes the carboxy terminus of both CDK11^p110^ and CDK11^p58^ isoforms; however, because CDK11^p58^ is typically only detected in cells transitioning from G2 to mitosis cell cycle phases, the vast majority of CDK11 detected in tissue array samples represents the CDK11^p110^ isoforms. IHC CDK11 staining specificity was verified by comparing results using two different commercial antibodies on human xenograft tumor tissues (data not shown). Upon staining of the tissue microarrays, CDK11 signal was observed in both malignant and normal tissue (Figure 1C). Unlike cells proliferating in culture, cells in normal breast tissue are less likely to be actively proliferating; however, the CDK11^p110^ isoforms have function in nondividing cells as well as actively proliferating cells, and thus were detected in normal breast epithelial cells as well as malignant cells. CDK11 protein expression in the breast tissues, as measured by IHC, was evaluated; the median score in TNBC was 7.5 (range 5.0 to 9.0) and was statistically significantly greater than the score in normal breast tissue (3.5 (range 1.8 to 7.5)) (Figure 1D). Overall, CDK11 staining was predominantly nuclear in both normal and TNBC tissues, with higher intensity and higher percentage of positive cells in TNBC (*P* <0.0001). The higher level of CDK11 protein signal in human TNBC observed here compared with normal breast is in agreement with the shift from moderate expression in breast glandular and myoepithelial cells to moderate–strong expression in breast cancer as documented on the Human Protein Atlas website [62,63].

IHC results for increased CK2α protein expression, especially in the nucleus, and correlation with metastatic risk in human breast cancer tissues have been published previously [44,64], and specific staining for CK2αα′ in human TNBC tissues was not performed here.

### Expression of CDK11 and CK2 protein complex genes in human breast cancer subtypes

**Figure 2:**
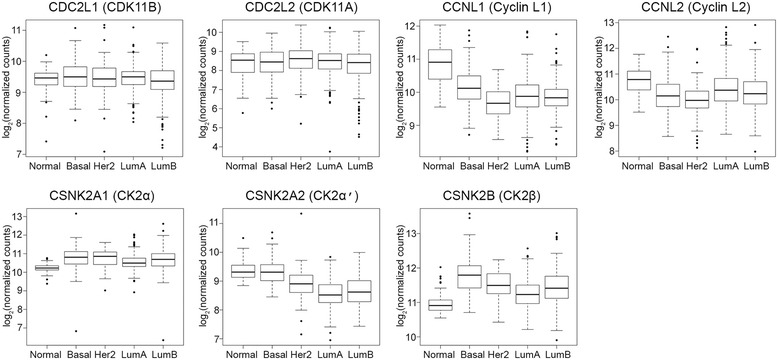
**RNA expression levels in normal breast and breast cancer subtypes.** Normalized RNAseq read count data for PAM50 breast cancer subtypes and normal breast from The Cancer Genome Atlas were analyzed for CDK11 and CK2 protein complex genes as shown above each plot. Box, first to third (Q1 to Q3) quartiles; line inside box, median; whiskers, 1.5 maximum interquartile range. Normal, *n* = 95; basal, *n* = 141; Her2, *n* = 67; LumA, *n* = 421; LumB, *n* = 192. CDK, cyclin-dependent kinase; CK2, casein kinase 2; Her2, human epidermal growth factor receptor 2; LumA, luminal A; LumB, luminal B.

Given the increased intensity of CDK11 protein signal in the TNBC tissues, we examined the mRNA transcript expression levels for CDK11 (*CDC2L1*, *CDC2L2*) using The Cancer Genome Atlas RNAseq data. We performed the same analysis for cyclin L1 (*CNCL1*), cyclin L2 (*CNCL2*), CK2α (*CSNK2A1*), CK2α′ (*CSNK2A2*), and CK2β (*CSNK2B*). We used the PAM50 subtypes to compare transcript expression in basal-subtype breast cancer with normal breast tissue and with the other breast cancer subtypes. The data are summarized in Figure 2 and Table 3. CDK11A showed no significant differences in mRNA expression compared with normal breast and with other breast cancer subtype tissues, and CDK11B expression in the basal subtype demonstrated a significant increase in mRNA expression relative to luminal B. Cyclin L1 mRNA expression was significantly lower in basal than normal tissue, but significantly higher than all other breast cancer subtypes. Cyclin L2 transcript expression was also significantly lower in basal breast cancer than in normal breast. CK2α mRNA expression was significantly higher in the basal subtype than in normal tissue and the luminal A subtype. CK2α′ transcript levels were the same in the basal subtype as in normal tissue and significantly higher in the basal subtype than in HER2, luminal A, and luminal B cancers. Finally, CK2β mRNA expression in basal breast cancer was significantly higher than normal tissue and all other breast cancer subtypes. In these RNAseq analyses, the basal subtype was used as a surrogate for TNBC; additionally, we found highly similar expression patterns when comparing TNBC with all other biomarker breast cancers using microarray datasets found on Oncomine [65] (data not shown).

**Table 3:** **mRNA expression levels in normal and breast cancer subtypes**

**Gene**	**Subtype or normal** ^**a**^	**Log**_**2**_ **(normalized counts)** ^**b**^	**Comparison with basal** ^**c**^
*CDC2L1*	Normal	9.39 ± 0.38	0.90
	Basal	9.51 ± 0.5	N/A
	Her2	9.45 ± 0.61	1
	Luminal A	9.46 ± 0.4	1
	Luminal B	9.33 ± 0.57	**0.049**
*CDC2L2*	Normal	8.35 ± 0.76	−^**d**^
	Basal	8.37 ± 0.79	N/A
	Her2	8.49 ± 0.84	–
	Luminal A	8.44 ± 0.69	–
	Luminal B	8.24 ± 0.95	–
*CCNL1*	Normal	10.82 ± 0.58	**1.37 × 10** ^**−14**^
	Basal	10.15 ± 0.56	N/A
	Her2	9.7 ± 0.49	**9.68 × 10** ^**−7**^
	Luminal A	9.87 ± 0.55	**2.17 × 10** ^**−5**^
	Luminal B	9.85 ± 0.49	**2.10 × 10** ^**−5**^
*CCNL2*	Normal	10.79 ± 0.48	**1.90 × 10** ^**−11**^
	Basal	10.2 ± 0.71	N/A
	Her2	10 ± 0.73	1
	Luminal A	10.38 ± 0.7	0.21
	Luminal B	10.28 ± 0.7	1
*CSNK2A1*	Normal	10.22 ± 0.2	**5.50 × 10** ^**−15**^
	Basal	10.74 ± 0.64	N/A
	Her2	10.75 ± 0.51	1
	Luminal A	10.53 ± 0.39	**9.20 × 10** ^**−3**^
	Luminal B	10.68 ± 0.61	1
*CSNK2A2*	Normal	9.36 ± 0.3	1
	Basal	9.3 ± 0.39	N/A
	Her2	8.92 ± 0.56	**3.89 × 10** ^**−5**^
	Luminal A	8.56 ± 0.45	**2.06 × 10** ^**−49**^
	Luminal B	8.64 ± 0.47	**2.99 × 10** ^**−33**^
*CSNK2B*	Normal	10.96 ± 0.28	**3.03 × 10** ^**−38**^
	Basal	11.78 ± 0.5	N/A
	Her2	11.52 ± 0.39	**1.91 × 10** ^**−3**^
	Luminal A	11.25 ± 0.42	**4.04 × 10** ^**−22**^
	Luminal B	11.43 ± 0.54	**8.24 × 10** ^**−8**^

Her2, human epidermal growth factor receptor 2; N/A, not applicable. ^a^Normal, *n* = 95; basal, *n* = 141; Her2, *n* = 67; luminal A, *n* = 421; luminal B, *n* = 192. ^b^RNAseq data from The Cancer Genome Atlas expressed as mean ± standard deviation. ^c^Comparison with basal subtype, *P* value with Bonferroni correction; significant *P* values in bold type. ^d^Analysis of variance comparing CDC2L2 expression across subtypes revealed no significant differences, so no pairwise *t* tests were performed.

### siRNA-mediated downregulation of CDK11 and CK2 in breast cancer cells induces death signaling, decreases cell viability, and inhibits clonal survival

**Figure 3:**
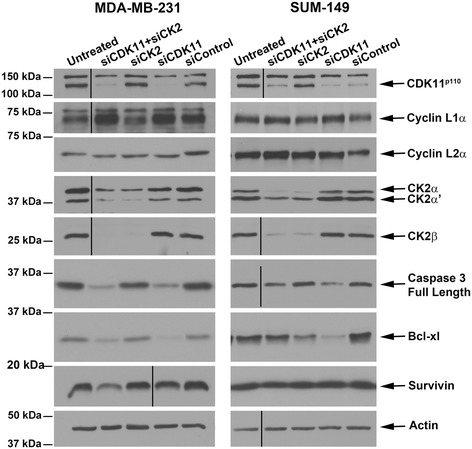
**Immunoblot analyses following small interfering RNA-mediated downregulation of CDK11 and CK2 in breast cancer cells.** Immunoblot analysis of MDA-MB-231 and SUM-149 cell lysates following small interfering RNA (siRNA) transfection. Transfected siRNAs are indicated above the blots, proteins detected are indicated on the right side of the blots. CDK11^p110^, cyclin L1α, cyclin L2α, and CK2αα′β lysates are 72 hours post transfection; caspase 3, Bcl-xL, and survivin lysates are 96 hours post transfection. Actin signal was used as the loading control. CDK, cyclin-dependent kinase; CK2, casein kinase 2.

We used siRNA sequences previously validated for both CDK11 gene transcripts, located 3′ to the CDK11^p58^ internal ribosomal entry site, and for both CK2α and CK2α′ catalytic subunit gene transcripts to test the effects of downregulation of CK2 alone, CDK11 alone, or combined CK2 and CDK11 in breast cancer cells [2,30]. Transfection of siCDK11 efficiently reduced CDK11^p110^ protein expression in MDA-MB-231 and SUM-149 cells by 72 hours post transfection (Figure 3). CDK11^p58^ was not detectable in these asynchronously growing cells. Loss of CDK11 expression did not have a consistent effect on cyclin L1α or cyclin L2α expression levels; however, downregulation of CDK11 resulted in faster migration of the cyclin L2α protein. We also observed that transfection of cultured cells frequently caused increased detection of cyclin L1α protein. Transfection of siCK2 significantly reduced expression of CK2α and CK2α′ at 72 hours in MDA-MB-231 and SUM-149 cells; concomitantly, CK2β expression levels were also significantly reduced (Figure 3). Interestingly, loss of CK2 also resulted in altered cyclin L2α migration. Loss of CK2 and/or CDK11 expression resulted in modulation of cell death associated gene expression at 96 hours post transfection. Reduced detection of full-length caspase 3 protein was observed (Figure 3), and activated caspase 3 cleavage products were also sometimes detected (data not shown). Decreased full-length lamin A/C and appearance of cleaved lamin A/C products were also observed (data not shown). Loss of Bcl-xL expression was more evident with knockdown of CDK11 than CK2 in both cell lines, whereas slight loss of survivin expression was mainly seen following combined kinase knockdown. Quantitative analysis of protein expression is summarized in Table 4.

**Table 4:** **Protein expression levels following small interfering RNA transfection**

	**siRNA**	**CDK11**	**Cyclin L1α**	**Cyclin L2α**	**CK2α**	**CK2α** **′**	**CK2β**	**Caspase 3 – full length**	**Bcl-xL**	**Survivin**
MDA-MB-231	siCDK11/CK2	0.09 ± 0.02	3.07 ± 1.56	0.73 ± 0.53	0.07 ± 0.08	0.05 ± 0.06	0.03 ± 0.03	0.45 ± 0.17	0.40 ± 0.38	0.73 ± 0.53
		*P* <0.0001			*P* <0.0001	*P* = 0.001	*P* <0.0001			
	siCK2	0.98 ± 0.17	1.82 ± 0.67	0.78 ± 0.45	0.07 ± 0.07	0.05 ± 0.06	0.06 ± 0.04	0.85 ± 0.57	0.62 ± 0.10	0.85 ± 0.33
					*P* <0.0001	*P* = 0.001	*P* <0.0001			
	siCDK11	0.04 ± 0.07	2.38 ± 0.18	0.91 ± 0.49	0.88 ± 0.17	1.13 ± 0.18	1.05 ± 0.15	0.57 ± 0.23	0.24 ± 0.05	0.79 ± 0.21
		*P* <0.0001							*P* = 0.022	
	siControl	0.90 ± 0.04	1.77 ± 0.31	1.44 ± 0.62	1.09 ± 0.10	0.98 ± 0.26	1.14 ± 0.04	0.99 ± 0.07	0.93 ± 0.32	1.02 ± 0.20
SUM-149	siCDK11/CK2	0.44 ± 0.04	1.05 ± 0.12	1.30 ± 0.48	0.06 ± 0.05	0.27 ± 0.10	0.25 ± 0.14	0.45 ± 0.17	0.31 ± 0.21	0.77 ± 0.33
		*P* <0.0001			*P* <0.0001	*P* = 0.0004	*P* = 0.001	*P* = 0.036	*P* = 0.034	
	siCK2	0.86 ± 0.13	0.93 ± 0.18	1.31 ± 0.52	0.08 ± 0.07	0.31 ± 0.23	0.14 ± 0.07	0.55 ± 0.34	0.73 ± 0.12	1.02 ± 0.10
					*P* <0.0001	*P* = 0.0007	*P* = 0.0004			
	siCDK11	0.40 ± 0.16	1.04 ± 0.05	1.00 ± 0.19	1.19 ± 0.14	0.96 ± 0.12	0.77 ± 0.26	0.62 ± 0.23	0.54 ± 0.31	0.98 ± 0.18
		*P* <0.0001								
	siControl	0.89 ± 0.12	0.89 ± 0.26	0.90 ± 0.87	1.36 ± 0.27	1.05 ± 0.29	0.88 ± 0.17	0.92 ± 0.24	1.08 ± 0.30	0.89 ± 0.22

All values normalized to actin expression and expressed relative to untreated cells as mean ± standard deviation. *P* values are listed underneath the corresponding protein expression value for comparison with untreated cells if *P* <0.05. CDK, cyclin-dependent kinase; CK2, casein kinase 2; siRNA, small interfering RNA.

The specificity of the CDK11 and CK2 siRNAs for targeting the intended genes was examined by quantitative real-time RT-PCR. MDA-MB-231 and SUM-149 cells were transfected with 30 nM siRNAs (siCDK11, siCK2, siControl), cells were collected at 24 hours post transfection, and quantitative real-time RT-PCR was performed. CDK11 mRNA was decreased to 59% in both cell lines by siCDK11 transfection (Table 5). Strong downregulation of CK2α and CK2α′ mRNAs to less than 20% was observed in both TNBC cell types. Neither siCK2 nor siCDK11 transfections resulted in off-target decreased expression of CDK11 or CK2αα′ mRNAs, respectively (Table 5). Decreased protein expression for the targeted mRNAs was observed at 48 hours (data not shown).

**Table 5:** **mRNA expression levels in small interfering RNA transfected cells**

	**MDA-MB-231**		**SUM-149**	
	**siCDK11**	**siCK2**	**siCDK11**	**siCK2**
CDK11	0.59	1.36	0.59	0.98
CK2α	2.09	0.15	1.37	0.13
CK2α′	1.48	0.19	1.18	0.16

mRNA expression at 24 hours normalized to HPRT-1 and expressed relative to siControl; mean of duplicate wells. si, small interfering.

**Figure 4:**
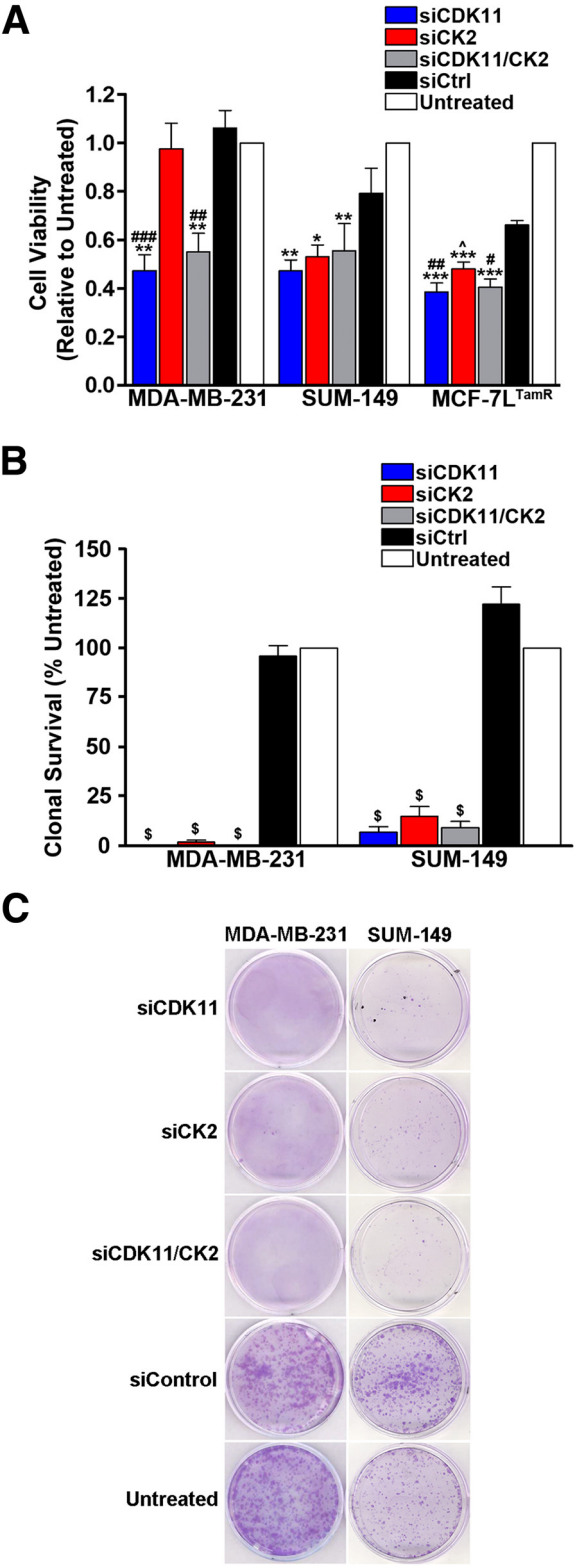
**Small interfering RNA-mediated downregulation of CDK11 and CK2 in breast cancer cells decreases cell viability and inhibits clonal survival. (A)** Breast cancer cells were transfected with 30 nM single small interfering RNA (siRNA) or 15 nM each of the two siRNAs combined as indicated. After 96 hours, cell viability was determined relative to the untreated cells. Means ± standard errors (SEs) are presented. *P <0.05. **P <0.01, ***P <0.001 relative to untreated. ^^^ *P* = 0.055, ^#^ *P* <0.05, ^##^ *P* <0.01, ^###^ *P* <0.001 relative to siCtrl. **(B)** Triple-negative breast cancer (TNBC) cells were transfected twice with 30 nM single siRNAs or 15 nM each of the two siRNAs combined as indicated and as described in Materials and methods. Seven days after the second transfection, cell colonies were stained and counted. Means ± SE are presented. ^$^ *P* <0.0001 relative to siCtrl and untreated. **(C)** Representative crystal violet stained colonies on 35 mm plates 7 days after the second siRNA transfection as described in (B). Cell lines are indicated above the plate images and siRNA transfections are indicated to the left of the plate images. CDK, cyclin-dependent kinase; CK2, casein kinase 2; si, small interfering.

The viability of cells following a single transfection of siCDK11, siCK2, or combined siCK2/siCDK11 was measured in MDA-MB-231, SUM-149, and MCF-7^TamR^ cell lines using the CellTiter 96® Aqueous One assay. Transfected cells were plated into wells coated with Matrigel, tenascin-C, and fibronectin to more closely mimic the *in vivo* tumor cell environment. Significant reductions in cell viability were observed for all three transfection types for SUM-149 and MCF-7^TamR^ cells; whereas significant reductions in viability were observed in MDA-MB-231 cells for siCDK11 and siCK2/siCDK11, but not siCK2 alone (Figure 4A). Given that visual observation of cells and immunoblot data suggested that MDA-MB-231 cells were, in fact, dying due to siCK2 transfection, we decided to use a different assay to evaluate cell survival following transfection of the different siRNAs. The clonal survival assay was employed in which MDA-MB-231 and SUM-149 cells were transfected twice with the siRNA combinations – once on day 1 and once on day 4. Twenty-four hours after the second transfection, the cells were collected using trypsin and plated in triplicate at 2,000 cells per 35 mm plate. After 7 days of incubation, the cell colonies were stained with crystal violet and counted. Downregulation of CDK11, CK2, or combined CDK11/CK2 protein expression resulted in highly significant loss of cell survival in SUM-149 cells and almost complete loss of cell survival in MDA-MB-231 cells (Figure 4B). Representative crystal violet stained colonies are shown in Figure 4C.

### Tenfibgen nanocapsules enter cultured and xenograft triple-negative breast cancer cells

**Figure 5:**
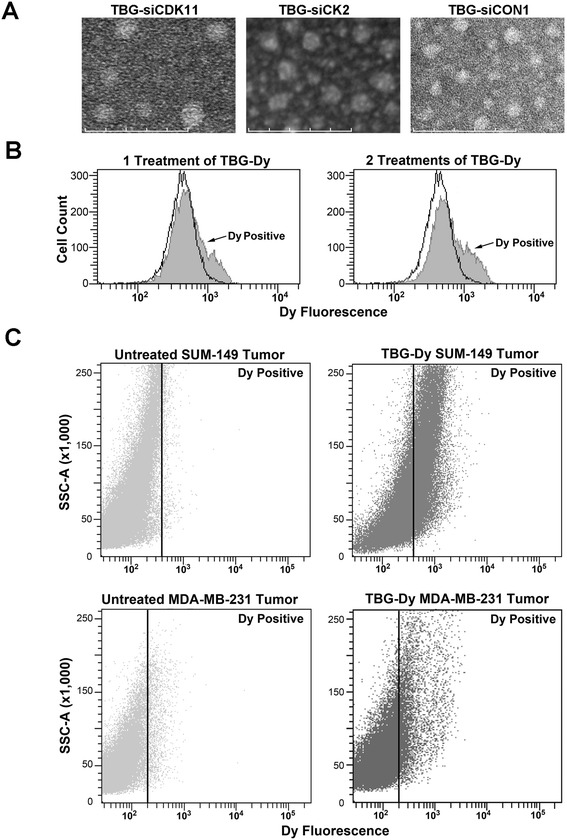
**Nanocapsule morphology and uptake efficiency in cultured cells and in xenograft tumors. (A)** Transmission electron micrographs of TBG-siCDK11, TBG-siCK2, and TBG-siCON1 nanocapsules used for *in vivo* studies. Scale bar: 100 nm. **(B)** MDA-MB-231 fluorescence-activated cell sorting (FACS) analysis for dysprosium (Dy) in untreated cells (black outline) and in TBG-Dy treated cells (gray). The number of TBG-Dy treatments is indicated above the graphs. **(C)** FACS analysis of xenograft tumor cells from untreated mouse (left panels) and from intravenous TBG-Dy-treated mouse (right panels). The identity of the tumor type is indicated above each panel. The position of the gate set to define Dy-positive cells is shown as a black line with Dy-positive events to the right of the line. CDK, cyclin-dependent kinase; CK2, casein kinase 2; si, small interfering; SSC, side scatter; TBG, tenfibgen.

To evaluate the *in vivo* effects on tumor growth of treatment with siCDK11 or siCK2, we employed a unique sub-50 nm size (that is, <50 nm) TBG-based nanocapsule that is capable of protected intracellular delivery of siRNA cargos in a malignant cell-specific manner [1-3]. Figure 5A and Table 6 define the physical characteristics of the TBG nanocapsules used in the therapeutic treatment studies. For the purpose of quantifying nanocapsule uptake, we used TBG nanocapsules containing a dysprosium dextran cargo. Dy is a fluorescent lanthanide element. To establish the feasibility of FACS analysis for Dy, MDA-MB-231 and SUM-149 cells were grown on combined tenascin-C and fibronectin matrix, treated in culture with TBG-Dy nanocapsules, and collected 1 day later for FACS analysis of the Dy content. Untreated cells were used to establish the analysis gate. Dy-positive cells totaled 14.4% in the MDA-MB-231 cells (Figure 5B, left panel and Table 7) and 13.1% in the SUM-149 cells (Table 7). Additionally, MDA-MB-231 cells were treated once in the morning and again 12 hours later to evaluate whether further uptake could be achieved. A 1.49-fold increase in Dy-positive cells was achieved by a second treatment (Figure 5B, right panel and Table 7).

**Table 6:** **Nanocapsule characteristics and information**

**Shell ligand**	**Particle size (nm)** ^**a**^	**Zeta potential (meV)** ^**b**^	**Morphology** ^**c**^	**Cargo** ^**d**^	**Sequence** ^**e**^
Tenfibgen	27.7 ± 4.6	−7.4 ± 4.2	Uniform, single capsules	siCDK11	5′-gagcgagcagcagcgugugdTdT-3′
27 kDa					
Tenfibgen	20.7 ± 5.2	−1.7 ± 2.4	Uniform, single capsules	siCK2	5′-auacaacccaaacuccacaudTdT-3′
27 kDa					
Tenfibgen	16.2 ± 2.4	−7.8 ± 4.2	Uniform, single capsules	siCON1	5′-uagcgacuaaacacaucaauudTdT-3′
27 kDa					

CDK, cyclin-dependent kinase; CK2, casein kinase 2; si, small interfering; TEM, transmission electron microscopy. ^a^Mean ± standard deviation of the average elliptical diameter determined from TEM micrographs measuring at least 20 nanocapsules. ^b^Average surface charge measured by dynamic light scattering from two different preparations across a 20 V potential in 1 mM KCl at 2 μg/ml. Data shown as the mean ± standard error of 15 independent measurements. ^c^Morphology of all nanocapsules determined by visual atomic force microscopy and TEM observation as uniform, single capsules. ^d^TBG-siCDK11, siCK2, and siCON1 encapsulation efficiencies mean of 54.0%, 86.8%, and 69.2%, respectively, observed by Burton analysis relative to unencapsulated siRNA. ^e^Sense strand sequence.

**Table 7:** **Fluorescence-activated cell sorting analysis of TBG-Dy uptake**

**Cell line or tumor**	**Treatment**	**Dy-positive (%)**
MDA-MB-231	Untreated	1.9
MDA-MB-231	TBG-Dy 1×	14.4
MDA-MB-231	TBG-Dy 2×	21.5
SUM-149	Untreated	0.9
SUM-149	TBG-Dy	13.1
SUM-149 tumor 1	Untreated	1.2
SUM-149 tumor 2	i.v. TBG-Dy	36.0
SUM-149 tumor 3	i.v. TBG-Dy	37.1
SUM-149 tumor 4	i.v. TBG-Dy	28.5
MDA-MB-231 tumor 1	Untreated	1.7
MDA-MB-231 tumor 2	i.v. TBG-Dy	11.0

Dy, dysprosium; i.v., intravenous; TBG, tenfibgen.

Having established TBG-Dy FACS methodology, we wished to quantitate the delivery of TBG nanocapsules to tumor cells *in vivo* after one intravenous treatment. Mice carrying MDA-MB-231 or SUM-149 mammary pad xenograft tumors were injected via the tail vein with TBG-Dy nanocapsule. After 24 hours, tumors were collected from the TBG-Dy-injected mice as well as non-injected control mice and subjected to dissociation. FACS analysis for Dy signal was performed, using the naive tumor cells to establish the gate (Figure 5C, left panels). After just one injection, an average of 33.9 ± 4.7% of the SUM-149 tumor cells was Dy-positive and 11.0% of the MDA-MB-231 tumor cells were positive for Dy signal (Figure 5C, right panels and Table 7).

### Treatment with TBG-siCDK11 or TBG-siCK2 nanocapsules causes MDA-MB-231 xenograft tumor shrinkage and loss of proliferation

**Figure 6:**
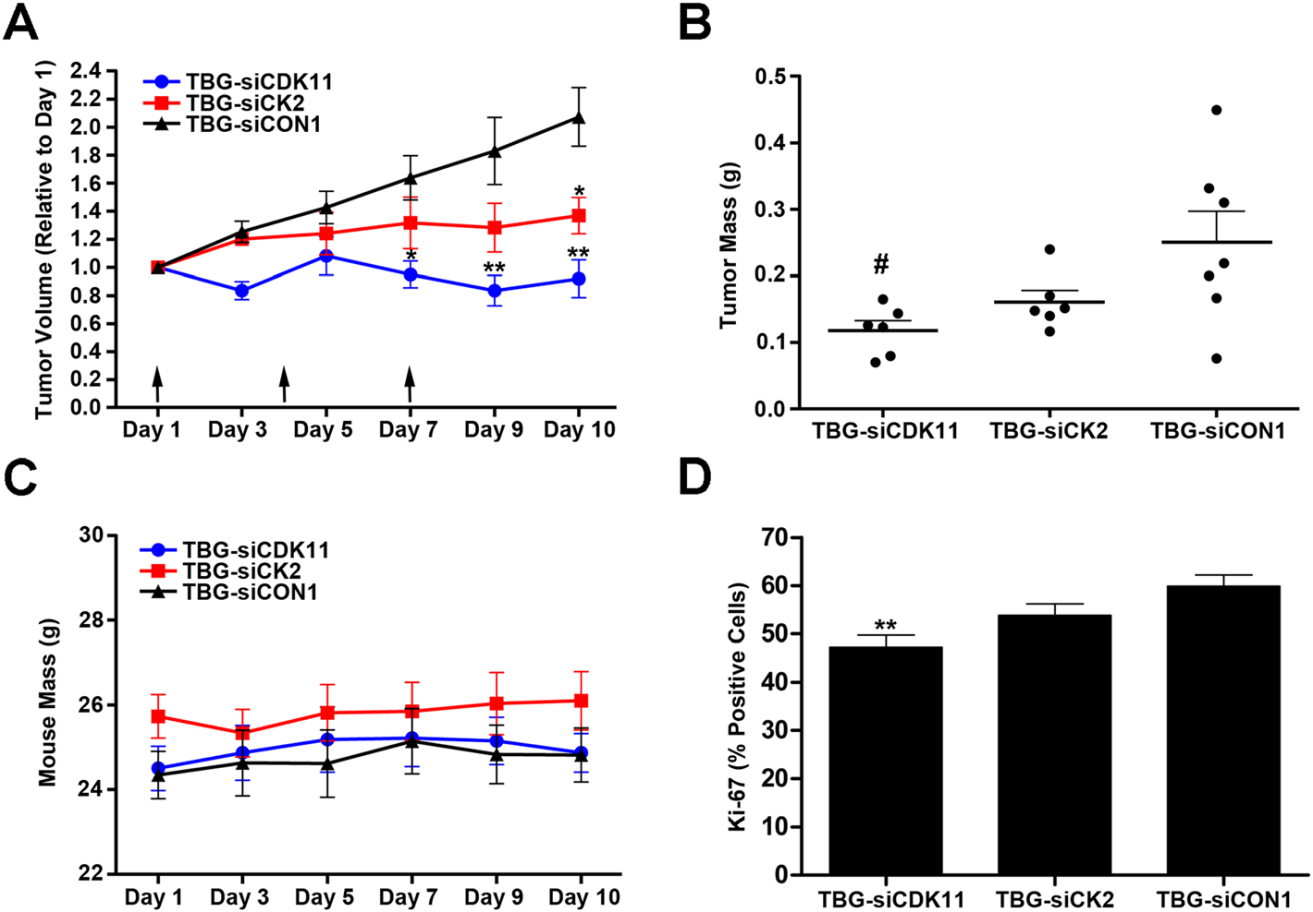
**Therapeutic effects of TBG-siCDK11 and TBG-siCK2 treatment in MDA-MB-231 xenograft tumors. (A)** Primary tumor volumes are shown following intravenous treatments at 0.01 mg/kg TBG-siCDK11, TBG-siCK2, or TBG-siCON1 on days 1, 4 and 7 (indicated by arrows). Means ± standard errors (SEs) are presented (TBG-siCDK11 and TBG-siCK2, *n* = 6; TBG-siCON1, *n* =7). *P <0.05, **P <0.01. **(B)** Primary tumor masses are shown following treatments as described in (A). Thick line, mean; thin bar, SE (TBG-siCDK11 and TBG-siCK2, *n* = 6; TBG-siCON1, *n* = 7). ^#^ *P* <0.05. **(C)** Masses of the mice throughout the study for each treatment group. Means are presented and error bars represent the SE. **(D)** Percentage of Ki-67-positive cells was analyzed as described in Materials and methods and is shown graphically. Least-squares means are presented and error bars represent SE. **P <0.01. CDK, cyclin-dependent kinase; CK2, casein kinase 2; si, small interfering; TBG, tenfibgen.

To demonstrate specific efficacy of CDK11 or CK2 targeting *in vivo* via TBG siRNA nanocapsules, we performed an acute response study in which nude mice carrying TNBC MDA-MB-231 flank tumors were treated three times by tail vein injection with 0.01 mg/kg of TBG-siCDK11, TBG-siCK2, or TBG-siCON1 (a control nontargeting siRNA [57]). This dose and schedule was chosen based on studies performed in prostate cancer models (unpublished data). Tumors were collected 10 days after initiation of treatment, and showed significant reduction of tumor volume below the starting volume for TBG-siCDK11 (*P* = 0.001) and just above the starting volume for TBG-siCK2 (*P* = 0.026, Figure 6A). Tumor weights were also reduced in TBG-siCDK11-treated (*P* = 0.030) and TBG-siCK2-treated mice compared with TBG-siCON1-treated mice (Figure 6B). No significant changes in mouse weights were observed following treatment with any of the nanocapsules (Figure 6C). Finally, tumor proliferation was evaluated by Ki-67 IHC and both TBG-siCDK11-treated (*P* = 0.004) and TBG-siCK2-treated tumors demonstrated reduced proliferation rates (Figure 6D).

### Treatment with TBG-siCDK11 or TBG-siCK2 induces RNA-induced silencing complex cleavage of the relevant mRNA transcripts in TNBC orthotopic xenograft tumors

**Figure 7:**
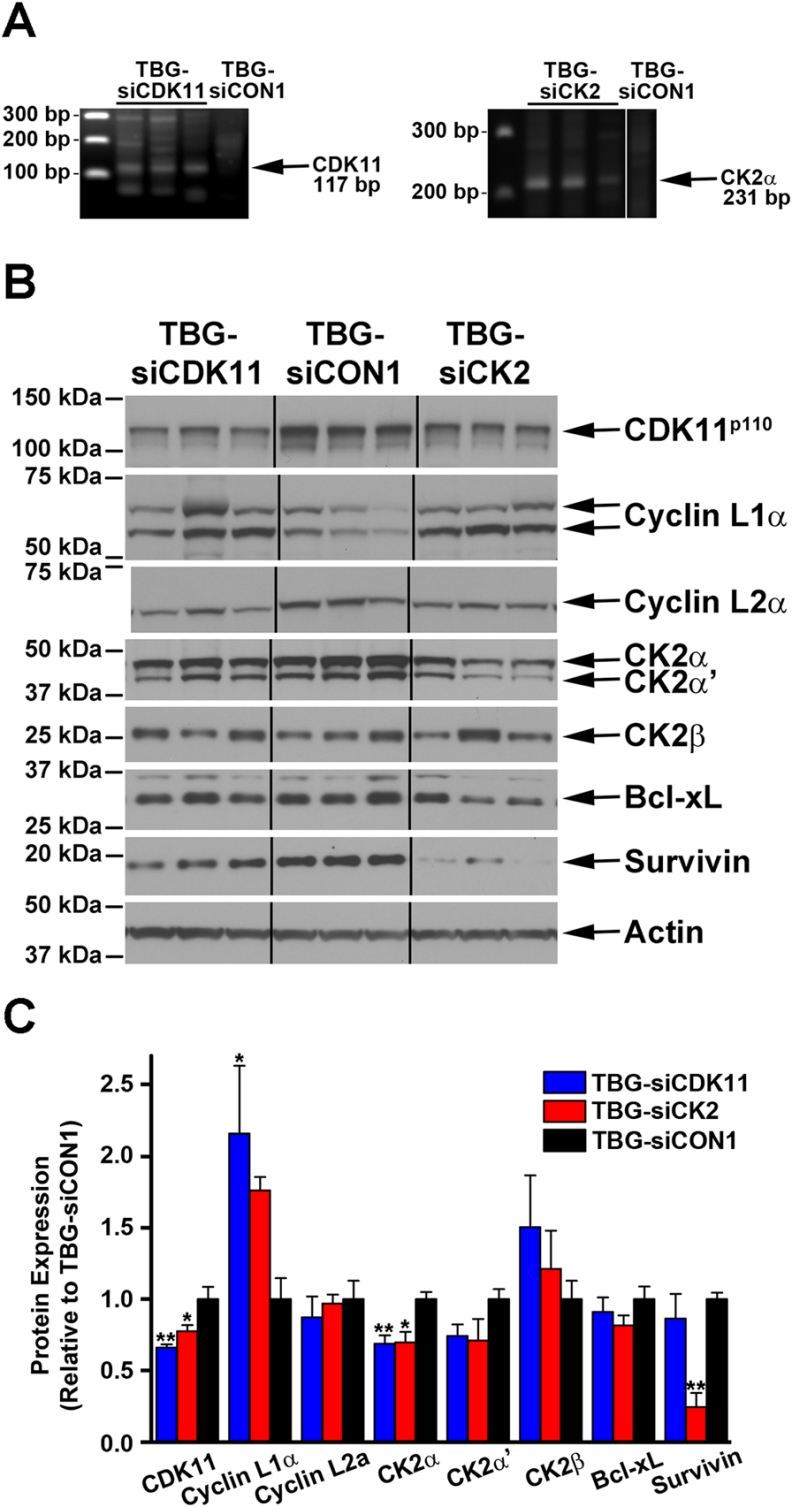
**Analysis for RNA-induced silencing complex cleavage products in treated tumors and immunoblot analysis for target protein complexes and death signals in primary tumors. (A)** Total RNA was isolated from tumor tissue and used for 5′ ligation-mediated RACE to determine whether RNA-induced silencing complex (RISC)-mediated cleavage of the transcript occurred. The predicted RACE products are indicated to the right, the size (base pairs (bp)) of the DNA standards shown on the left, and the treatment administered indicated above the lanes. **(B)** Immunoblot analysis of MDA-MB-231 day 10 tumor lysates following intravenous treatments of 0.01 mg/kg TBG-siCDK11, TBG-siCK2, or TBG-siCON1 as indicated above the blots. The signals for three mice per group are shown and the proteins detected are indicated on the right side of the blots. Actin signal was used as the loading control. **(C)** The protein signals from all mice in each treatment group were quantitated by densitometry using ImageJ software (National Institutes of Health Bethesda, MD, USA). Data presented as mean ± standard error. *P <0.05, **P <0.01. CDK, cyclin-dependent kinase; CK2, casein kinase 2; si, small interfering; TBG, tenfibgen.

RNA from TBG-siCDK11-treated and TBG-siCK2-treated tumors was analyzed using the modified 5′ RACE technique to detect potential cleavage products. Because there was not enough MDA-MB-231 TBG-siCDK11-treated tumor remaining for this analysis, we used SUM-149 mammary pad tumors from another study. The data indicated that RNA-induced silencing complex (RISC) cleavage products were detected for CDK11 and for CK2α mRNAs in tumors specifically following TBG-siCDK11 and TBG-siCK2 treatments, respectively (Figure 7A). No CDK11 or CK2 cleavage products were detected in TBG-siCON1-treated tumors. RISC cleavage products for CK2α were also detected in MDA-MB-231 xenograft tumors (data not shown). No CK2α′ cleavage products were detected in either SUM-149 or MDA-MB-231 TBG-siCK2-treated tumors.

### TBG-siCDK11-treated and TBG-siCK2-treated tumors show loss of target protein expression and induction of death signaling

Tumor lysates were subjected to immunoblot analysis to evaluate the effects of nanocapsule treatment on target gene expression (Figure 7B). TBG-siCDK11 treatment resulted in CDK11^p110^ protein expression reduced to 66% relative to TBG-siCON1 (*P* = 0.002), cyclin L1α increased to 216% (*P* = 0.040), and cyclin L2α reduced to 87% (Figure 7C). Cyclin L2α migration was slightly faster in the TBG-siCDK11-treated and TBG-siCK2-treated tumors, analogous to what was seen in cultured cells. TBG-siCK2 treatment resulted in CK2α protein expression reduced to 69% (*P* = 0.010), CK2α′ reduced to 74%, and CK2β increased to 121%. Unexpectedly, TBG-siCDK11-treated tumors showed reduced expression of CK2α (*P* = 0.008) and CK2α′, and TBG-siCK2-treated tumors showed reduced expression of CDK11^p110^ (*P* = 0.039). TBG-siCK2 treatment also increased cyclin L1α expression (Figure 7C). These data demonstrate reduced expression of targeted proteins, and some possible cross-influence between the two protein kinase complexes.

The tumor lysates were further examined for effects on downstream death signals. Bcl-xL protein was slightly reduced to 91% and 81% following TBG-siCDK11 and TBG-siCK2 treatments, respectively, although neither were statistically significant (Figure 7C); no changes in caspase 3 protein expression were detected (data not shown). A small decrease to 86% was observed for survivin in TBG-siCDK11-treated tumors, whereas a significant decrease in survivin expression to 25% (*P* = 0.001) was seen in TBG-siCK2-treated tumors (Figure 7C).

## Discussion

This work represents the first report specifically investigating CDK11 and CK2 as targets for *in vivo* TNBC therapy. The significance of CDK11 as the proposed therapeutic target lies in the possibility of affecting both mitotically cycling and noncycling or quiescent malignant cells because the expression and functions of CDK11 isoforms affect both proliferating and quiescent cells. This dual consequence of targeting CDK11 adds unique functionality not observed by targeting family members such as CDK1, CDK2, CDK4, or CDK6. Thus, CDK11 downregulation would potentially overcome a known failure point in some existing cancer therapeutics, which is the lack of efficacy in quiescent cells [66,67]. The utility of targeting CDK11 is further supported by a recent investigation using intratumoral delivery of naked siRNAs in osteosarcoma xenograft tumors that demonstrated the efficacy of reduced CDK11 expression in this model [15]. The significance of CK2 as the proposed target is found in the extensive functions this master regulator performs in cells, and its critical involvement in the oncogenic phenotype [37,43]. Notably, increased expression of CK2α in the mouse mammary gland under control of the MMTV-LTR resulted in a transgenic mouse model of breast cancer [49]. Further, a recent report highlighted the anti-survival effects of targeting CK2 in breast cancer [68], and oral treatment of mice carrying BT-474 breast cancer xenografts with the CK2 inhibitor CX-4945 inhibited xenograft tumor growth [69]. Data presented here showed that knockdown of either of these kinases using siRNA technology was effective at inducing cell death in cultured cells as well as TNBC xenograft tumors. Although we focused primarily on TNBC in this work, CK2 and CDK11 targeting strategies are likely to be effective across the breast cancer subtypes; however, further investigation will be needed to verify this supposition.

Basing the choice of therapeutic target genes on those that demonstrate increased mRNA expression levels in cancer compared with normal tissue results in omission of some candidates. mRNA levels cannot consistently be used as surrogates for the corresponding protein expression levels; in fact, less than 50% of genes demonstrate correlation between RNA and protein expression levels [70,71]. Moreover, there is the added complication that loss of translational control of gene expression is a common event in solid tumors, including breast cancer [72]. Loss of translational control might account for the close correlation of CDK11 transcript and protein levels in the malignant cultured cell lines as compared with the nontransformed cells MCF-10A and MCF-12A. In normal tissue the CDK11 RNA levels are in the low range and the protein levels are in mid to high ranges, whereas in the cancer cell lines the RNA levels are in the mid range and more closely match the protein levels that are mid to high [62]. The RNAseq data we evaluated for CDK11 indicated an increase in basal breast cancer mRNA expression compared with the luminal B subtype and not compared with normal breast or other breast cancer subtypes; however, our IHC analysis of breast tissue showed an increase in CDK11 protein expression intensity in TNBC compared with normal breast. Future investigation into the post-transcriptional regulation of CDK11 in breast cancer may reveal further insights into the regulatory mechanisms in these malignancies.

In the past, although CK2 protein expression has been upregulated in all cancer types investigated to date [43,73], CK2 was not typically identified as a good target gene because microarray analysis did not show a significant increase in its RNA expression. More recently, the availability of RNAseq data has provided a new store of unbiased data on transcript expression. Protein atlas data show that, in normal tissue, CK2α RNA expression is low and the protein expression levels are mid to high [74]. CK2α expression in cancer cell lines shows mid-level RNA expression and mid to high levels for protein, demonstrating increased concordance of RNA and protein expression in cancer. Our RNAseq analyses here demonstrated increased CK2α and CK2β mRNA expression in TNBC compared with normal tissue, validating the utility of CK2 as a target in TNBC. While increased CK2α protein expression in overall breast cancer is established, the more specific roles of CK2α and CK2β expression at the protein level in TNBC are not yet defined [44,75].

Targeting CDK11 and CK2 in combination in cultured cells did not notably increase the induction of cell death over that observed targeting the kinases individually, so the combination was not tested *in vivo*. Additionally, no reduction in CK2αα′ mRNA at 24 hours or protein expression at 72 hours was observed when CDK11 was downregulated using siRNA in cultured cells, and the reverse for CDK11 expression levels in siCK2 transfected cells was also true. Interestingly, the treated tumor data here suggest some reciprocal influence between members of the CDK11 and CK2 protein complexes that was not seen in cultured cells. We observed loss of CDK11^p110^ upon downregulation of CK2αα′, and loss of CK2αα′ upon downregulation of CDK11^p110^. Cyclin L1 protein expression was increased following downregulation of either kinase. We have demonstrated previously that CDK11 and CK2 protein kinase complexes interact with one another within larger complexes regulating RNA transcription and splicing [9,23,24]. Further, we have shown that CK2 phosphorylates CDK11^p110^ [24]. Thus, it is possible that downregulation of one of these survival kinases *in vivo* is acting to impact the expression of the other kinase, contributing to the decreased survival of the TNBC cells. CK2 regulates survivin gene expression in cultured cells such that loss of CK2 activity decreases survivin expression [76,77]. The significant downregulation of survivin protein following TBG-siCK2 treatment, but not following TBG-siCDK11 treatment, suggests that downregulation of CK2 in the TBG-siCDK11-treated tumors possibly occurred too late in the acute response study to have markedly affected survivin expression at the time of tumor collection.

Detection of CDK11 mRNA cleavage products in TBG-siCDK11-treated tumors demonstrates that the siRNA is acting through the RISC/Argonaute 2 pathway. The siCDK11 siRNA sequence used in this experiment has 3/19 mismatches comparing the human CDK11A and CDK11B genes with the homologous mouse CDK11B (*Cdc2L1*) sequence; human siCDK11 is thus not likely to knockdown mouse CDK11 expression by Argonaute 2/RISC-based mechanisms, and the RACE products detected most probably derive from the human xenograft tumor cells. For TBG-siCK2-treated tumors, we were able to amplify CK2α but not CK2α′ mRNA cleavage products. The siCK2 siRNA sequence has 1/20 mismatches with human CK2α′, and this may explain why it is more difficult to detect CK2α′ cleavage product. We did not have enough tumor material after protein analyses to perform human-specific quantitative real-time PCR for the MDA-MB-231 treatment study; however, the specific detection of the mRNA cleavage products in both SUM-149 and MDA-MB-231 tumors from mice treated intravenously using the TBG-siCDK11 and TBG-siCK2 nanocapsules, respectively, suggests that the appropriate transcripts are targeted for reduction. The effect of TBG-siCDK11 and TBG-siCK2 treatment on target mRNA levels will be determined in a future study.

There is ongoing research to develop small molecule inhibitors for several CDKs; however, to our knowledge no specific inhibitor of CDK11 has emerged. Many inhibitors to CK2 have been developed, and the inhibitor CX-4945 – initially used in clinical trials as a single agent – is currently in clinical trials as a combinatorial agent with gemcitabine and cisplatin for treatment of cholangiocarcinoma. It is well documented that cancer cells frequently circumvent the effectiveness of small molecule inhibitors over time, thus necessitating the development of alternate strategies for cancer therapy [78,79]. Additionally, unlike small molecule inhibitors, targeting the expression of an individual protein using siRNAs provides a means to precisely impact its expression and activity, providing potential insight into novel interactions and/or pathways that are specific for that protein.

Our TBG nanocapsule containing siRNAs directed against either both CDK11 genes or against CK2α and CK2α′ provides protection to the nucleic acid during circulation and releases the siRNA within the cancer cells to downregulate the targeted gene expression. Malignant cell-directed treatment using the TBG nanoencapsulation technology enables targeting of survival genes without associated harm from downregulation of essential genes in normal cells [1,3]. Interestingly, reduction of less than 35% of either kinase in MDA-MB-231 tumors resulted in significant effects on tumor volume relative to controls after only three doses of the therapeutic agents. Further, treatment with TBG-siCDK11 also significantly decreased proliferative activity. These results demonstrate that moderate knockdown of either CDK11 or CK2 significantly impacts the growth and viability of TNBC tumors. Future work will focus on investigating the use of TBG-siCDK11 and TBG-siCK2 in various models of breast cancer as well as the interplay between CDK11 and CK2 and their potential co-regulation.

## Conclusions

CDK11 expression and CK2 expression are individually essential for breast cancer cell survival, including TNBC in culture and in a MDA-MD-231 xenograft model. Our data demonstrate that TBG nanoencapsulation of anti-CDK11 or anti-CK2 siRNAs is a highly specific means of successfully delivering the drug to malignant cells *in vivo*, and moderate knockdown of either kinase resulted in significant therapeutic benefit. We propose that CDK11 and CK2 serve as promising targets for further development of treatment in breast cancer.

## Abbreviations

ANOVA: analysis of variance
CDK: cyclin-dependent kinase
CK2: casein kinase 2
DMEM: Dulbecco’s modified Eagle’s medium
Dy: dysprosium
EDTA: ethylenediamine tetraacetic acid
FACS: fluorescence-activated cell sorting
FBS: fetal bovine serum
IHC: immunohistochemistry
PBS: phosphate-buffered saline
RISC: RNA-induced silencing complex
RNAi: RNA interference
siCDK11: small interfering RNA to cyclin-dependent kinase 11
siCK2: small interfering RNA to CK2αα′
siCON1: small interfering RNA to control siRNA CON1
siRNA: small interfering RNA
TBG: tenfibgen (carboxy-terminal fibrinogen globe domain of tenascin-C)
TNBC: triple-negative breast cancer
